# Gut microbiota dysbiosis is associated with hippocampal neuronal damage and behavioral alterations in APP/PS1 mice with high-salt diet

**DOI:** 10.3389/fmicb.2026.1884427

**Published:** 2026-09-11

**Authors:** Huanmin Zhang, Jinxin Kou, Pengfei Ge, Lingzhuan Gong, Wen Zhang, Yulang Fei

**Affiliations:** 1 Department of Clinical Rehabilitation, School of Medicine, Xi’an Siyuan University, Xi’an, China; 2 Department of Neurology, Shiyan Taihe Hospital, Affiliated Hospital of Hubei University of Medicine, Shiyan, China; 3 Department of Rehabilitation Medicine, Ordnance Industry General Hospital, Xi’an, China; 4 The First Affiliated Hospital of Nanyang Medical College, Nanyang, Henan Province, China; 5 Zhang Zhongjing Chinese Medical Research Institute, Nanyang Medical College, Nanyang, Henan Province, China

**Keywords:** Alzheimer’s disease, cognitive impairment, gut microbiota, high-salt diet, neuronal damage

## Abstract

**Introduction:**

High-salt diet (HSD) has been shown to influence cognition and emotional behavior in mice via gut microbiota modulation, yet its chronic effects in Alzheimer’s disease (AD) pathology remain poorly understood. This study aimed to investigate whether long-term HSD exacerbates cognitive and emotional deficits in APP/PS1 transgenic mice and to explore the underlying gut–brain axis mechanisms involving microbiota dysbiosis, peripheral and central inflammation, hippocampal neuronal integrity, and metabolic alterations.

**Methods:**

Six-month-old male APP/PS1 mice were randomly assigned to a normal diet (ND, 0.4% NaCl, *n* = 43) or high-salt diet (HSD, 8% NaCl, *n* = 41) for 6 months. Body weight, water intake, and blood pressure were monitored regularly (*n* = 10 per group). Behavioral phenotypes were assessed via open field, elevated plus maze, marble burying, light–dark box, and novel object recognition tests (*n* = 10). Hippocampal neuronal density was quantified by Nissl staining in CA1 and CA2, and dendritic complexity was evaluated by Golgi staining with Sholl analysis (*n* = 4). Gut microbiota composition was profiled by 16S rRNA sequencing (ND: *n*=13; HSD: *n* = 12), and inflammatory cytokine expression (TNF-α, IL-6, and IL-1β) in brain and liver was measured by RT-PCR and ELISA (*n* = 4). Hippocampal metabolomics was performed using LC–MS (ND: *n* = 8; HSD: *n* = 7). Correlation analyses integrated microbial, inflammatory, metabolic, and neuropathological data.

**Results:**

HSD significantly altered gut microbiota structure, as shown by reduced α-diversity, distinct β-diversity (PCoA), and differential abundance of taxa including Prevotellaceae, Rikenellaceae, and Ruminococcaceae (LEfSe). Concurrently, HSD elevated pro-inflammatory cytokine expression in both brain and liver, reduced neuronal density in hippocampal CA1/CA2, and impaired dendritic arborization. Behaviorally, HSD-treated mice exhibited aggravated emotional disorders (anxiety- and compulsive-like behaviors) and worsened cognitive impairment in novelty recognition. Hippocampal metabolomics revealed substantial shifts in amino acid, energy, and neurotransmitter metabolic pathways. Spearman correlation analyses demonstrated significant interconnections among specific gut microbial genera, inflammatory markers, neuronal damage indices, and differential metabolites.

**Discussion and conclusion:**

Long-term high-salt intake exacerbates cognitive decline and emotional disturbances in APP/PS1 mice, likely through a cascading gut–brain axis pathway: HSD-induced microbiota dysbiosis promotes peripheral and central inflammation, which in turn drives hippocampal neuronal damage and metabolic reprogramming. The strong correlations among microbial shifts, metabolic alterations, and neuropathological changes suggest that gut microbiota may serve as a critical mediator of HSD’s deleterious effects on AD-related brain functions. These findings provide new mechanistic insights into dietary risk factors in AD and highlight potential microbiota–metabolite targets for therapeutic intervention.

## 1 Introduction

Sodium chloride is an essential substance for the human body and plays a crucial role in maintaining vital bodily functions. The World Health Organization’s healthy diet guidelines recommend that daily salt intake should be less than 5 grams (Wang et al., 2024). A high-salt diet is a widespread phenomenon globally (Thout et al., 2019)，Statistical analysis of Chinese dietary habits shows that a high-salt diet has become a common dietary habit, particularly in the northwestern regions, where the average daily salt intake per capita is 12–18 grams (Liu et al., 2015; He et al., 2018). Accumulating animal evidence indicates that a high-salt diet can significantly alter the composition and function of the gut microbiota (Lei et al., 2023; Xu et al., 2025). Mouse studies have demonstrated that excessive salt intake decreases intestinal Lactobacillus abundance and suppresses butyrate synthesis (Miranda et al., 2018). Consistently, long-term mouse experiments confirmed that 22 weeks of high-salt feeding induced prominent shifts in the composition and diversity of gut microbiota (Tian et al., 2025).

Growing human-based research has depicted the baseline landscape of human intestinal microbial communities. The gut microbiota consists of bacteria, archaea, viruses, and fungi that colonize the gastrointestinal tracts of humans and other animals, playing a crucial role in maintaining host health and physiological functions (Kandari et al., 2024; Ronen et al., 2024). Bacteroidetes and Firmicutes constitute approximately 90% of the human gut microbiota (Ndeh and Gilbert, 2018; Ajith and Anita, 2025). In addition, members of the phyla Actinobacteria and Proteobacteria are also detected within the human gut (Ndeh and Gilbert, 2018).

Alterations in gut microbiota are associated with a variety of neurological disorders, including neurodegenerative diseases as well as emotional disorders such as anxiety and depression (Luna and Foster, 2015; Mincic et al., 2024; Chenghan et al., 2025; Mehta et al., 2025; Dagdeviren and Bozcal, 2025; Zhou et al., 2025). Numerous studies have shown that patients with Alzheimer’s disease (AD) exhibit reduced gut microbial diversity and altered abundances of certain bacterial species (Chen Q. et al., 2024; Kapoor et al., 2024; Kunevičius et al., 2025; Manfredi et al., 2025). It has been reported that high-salt diet also alternates gut microbiota, exacerbates cognitive deficits and neurovascular abnormalities in AD mice (Chen H. C. et al., 2024).

However, whether a high-salt diet induces hippocampal neuroinflammation and behavioral changes in mice via regulating gut microbiota remains unclear. The earlier study (Xu et al., 2025) mainly focused on SerpinA3n-mediated cerebral pathological alterations triggered by high-salt intake. The present research establishes an integrated gut-brain axis framework to address this question. Using 16S rRNA sequencing, we systematically characterize high-salt-evoked gut microbiota dysbiosis, alongside detection of peripheral and central inflammatory activation and identification of hippocampal neuronal injury. Furthermore, untargeted metabolomics reveals significant perturbations in hippocampal metabolites associated with amino acid, energy, and neurotransmitter pathways. Correlation analyses confirm the crosstalk between altered gut microbes, hippocampal metabolism, and neuronal damage. Collectively, our findings identify a previously unrecognized gut microbiota-metabolite pathway explaining how excessive salt intake exacerbates emotional disorders in AD-model mice, complementing the brain-centered mechanism reported in the prior literature.

## 2 Materials and methods

### 2.1 Animals

All experimental procedures were conducted in accordance with ethical guidelines to minimize the number of animals used and reduce their suffering, and were approved by the Animal Experiment Ethics Committee of the First Affiliated Hospital of Nanyang Medical College (Approval Number.: 2023007). APP/PS1 mice were housed under controlled environmental conditions (temperature 22 ± 2 °C, relative humidity 60%–70%, 12:12 h light–dark cycle) with free access to food and water. All 84 enrolled mice were included for data collection, and no mice were excluded throughout this study. Among them, 41 mice were assigned to the high-salt diet group (HS, 8% NaCl) and 43 mice to the control group (ND, 0.4% NaCl). The mice received dietary intervention for 6 months. And their body weight and water intake were regularly recorded.

### 2.2 Blood pressure

Blood pressure was measured using the CODA non-invasive blood pressure system (Kent Scientific, Torrington, Connecticut, USA) via tail cuff. Each mouse was placed in a restrainer, and a properly sized cuff was securely wrapped around its tail. Mice were kept on a heated platform for three measurements to ensure stable body temperature.

### 2.3 Behavioral test

All behavioral tests were performed during the dark phase, following a previously published experimental protocol (Ma et al., 2024).

Mice need to acclimate to the testing environment for 2 h before the test. To minimize cross-test effects, each test is conducted 24 h apart. Mice underwent a battery of behavioral assays, in the open-field test (40 cm × 40 cm × 30 cm arena, 6 min exploration), total travel distance, speed and zone-entry counts were quantified; the elevated plus-maze test recorded arm-specific entries, travel distance and general movement over 6 min; for the Y-maze test, total arm entries and spontaneous alternations were scored to calculate alternation percentage. In the marble-burying test, mice explored cages with 5-cm corncob bedding and 20 evenly distributed marbles for 30 min, and unburied marbles (<50% covered by bedding) were counted. The novel-object recognition test included a 6-min training phase with two identical objects, and short-term memory was assessed 2 h later by replacing one object with a novel object, with 6-min exploration recorded for discrimination index calculation. For the light–dark box test, mice began in the lit compartment and explored freely for 5 min; time spent in light compartment, compartment transitions and total distance were acquired and analyzed using Smart 3.0.

### 2.4 Real-time quantitative reverse transcription polymerase chain reaction (RT-qPCR)

mRNA was extracted from mouse hippocampal tissue using TRIzol (Thermo Fisher Scientific), and total RNA was extracted following the manufacturer’s instructions. cDNA was synthesized from the extracted RNA using the RevertAid First Strand cDNA Synthesis Kit. Quantitative PCR (qPCR) was performed using 2 × RealStar Fast Probe RT-qPCR premix, which included cDNA template, probes, and nuclease-free water. The thermal cycling conditions consisted of an initial denaturation step (95 °C for 2 min), followed by 35 cycles of denaturation (95 °C for 15 s) and annealing/extension (60 °C for 30 s).

All qPCR primers used in this study were designed in-house using NCBI Primer-BLAST, based on the reference sequences of the corresponding genes retrieved from the NCBI database, and their specificity was verified by BLAST alignment (Ye et al., 2012).

### 2.5 Thioflavin-S staining

S-plaques were incubated in a solution containing 0.5% thioflavin-S (dissolved in 50% ethanol) for 30 min. Subsequently, they underwent two 10-s differentiation treatments with 80% ethanol, followed by washing with 0.01 M PBS (Ma et al., 2024).

### 2.6 Histological staining

In the high-salt diet group (*n* = 4) and normal AD group (*n* = 4), neuronal density in the hippocampal CA1 and CA2 regions was evaluated using Nissl staining and Golgi staining (Ma et al., 2024; Xu et al., 2025). For Golgi staining, **s**taining was performed according to the manufacturer’s instructions using the kit (GMS80020.2, Jiangsu Aide Shen Biological Technology Co., Ltd.). The stained sections were observed and imaged under a light microscope at 600× magnification. Neurons were then analyzed by Sholl analysis using ImageJ software to assess dendritic complexity. Ten neurons were photographed from each hippocampus.

### 2.7 16S rRNA sequencing

16S rRNA sequencing: Cecal content samples were collected from both the high-salt diet group (*n* = 13) and the normal AD group (*n* = 12). After collection, the samples were immediately frozen and stored at −80 °C. RNA purity and concentration were assessed using a NanoDrop 2000 spectrophotometer (Thermo Fisher Scientific, USA), and RNA integrity was evaluated with an Agilent 2,100 Bioanalyzer (Agilent Technologies, Santa Clara, USA). Libraries were constructed using the TruSeq Stranded mRNA LT Sample Prep Kit (Illumina, USA) according to the manufacturer’s instructions. 16S rRNA gene amplicon sequencing and analysis were performed by Shanghai Oebiotech Bio Co., Ltd.

Microbiome bioinformatics analysis: Calculate α-diversity indices such as PD_whole_tree, Simpson, ACE, Chao1, observed_species, and Shannon. For β-diversity analysis, perform Bray-Curtis analysis using the Bray-Curtis distance matrix generated by QIIME software, and visualize it through Principal Coordinates Analysis (PCoA) to display structural changes in microbial communities between different groups. Differences in bacterial abundance values were calculated using Linear Discriminant Analysis (LDA) Effect Size (LEfSe). A LDA value greater than 2 was considered to indicate a significant difference (Tong et al., 2024).

### 2.8 Enzyme-linked immunosorbent assay

The concentrations of tumor necrosis factor-α (TNF-α), interleukin-6 (IL-6), interleukin-1β (IL-1β), and interleukin-10 (IL-10) in mouse serum and colon tissue homogenate supernatants were detected using the double-antibody sandwich enzyme-linked immunosorbent assay (ELISA). All ELISA experiments were operated strictly according to the manufacturer’s instructions (Beyotime Biotechnology, Shanghai, China). All data are presented as mean ± standard deviation.

### 2.9 Statistics

Normally-distributed data are expressed as mean ± SD, and non-normal data as median with interquartile range (IQR). Analyses were run in GraphPad Prism 9.5, with normality evaluated by the Shapiro–Wilk test. Two-group comparisons used unpaired Student’s t-test for parametric data and Mann–Whitney U test for non-parametric data. Gut-microbiota taxonomic differences were assessed by Wilcoxon rank-sum test, and Spearman correlation was computed via the OECloud platform. Statistical significance was defined as *p* < 0.05 (Xu et al., 2025).

## 3 Results

### 3.1 High-salt diet alters cognitive and anxiety-like behavioral in AD mice

**Figure 1:**
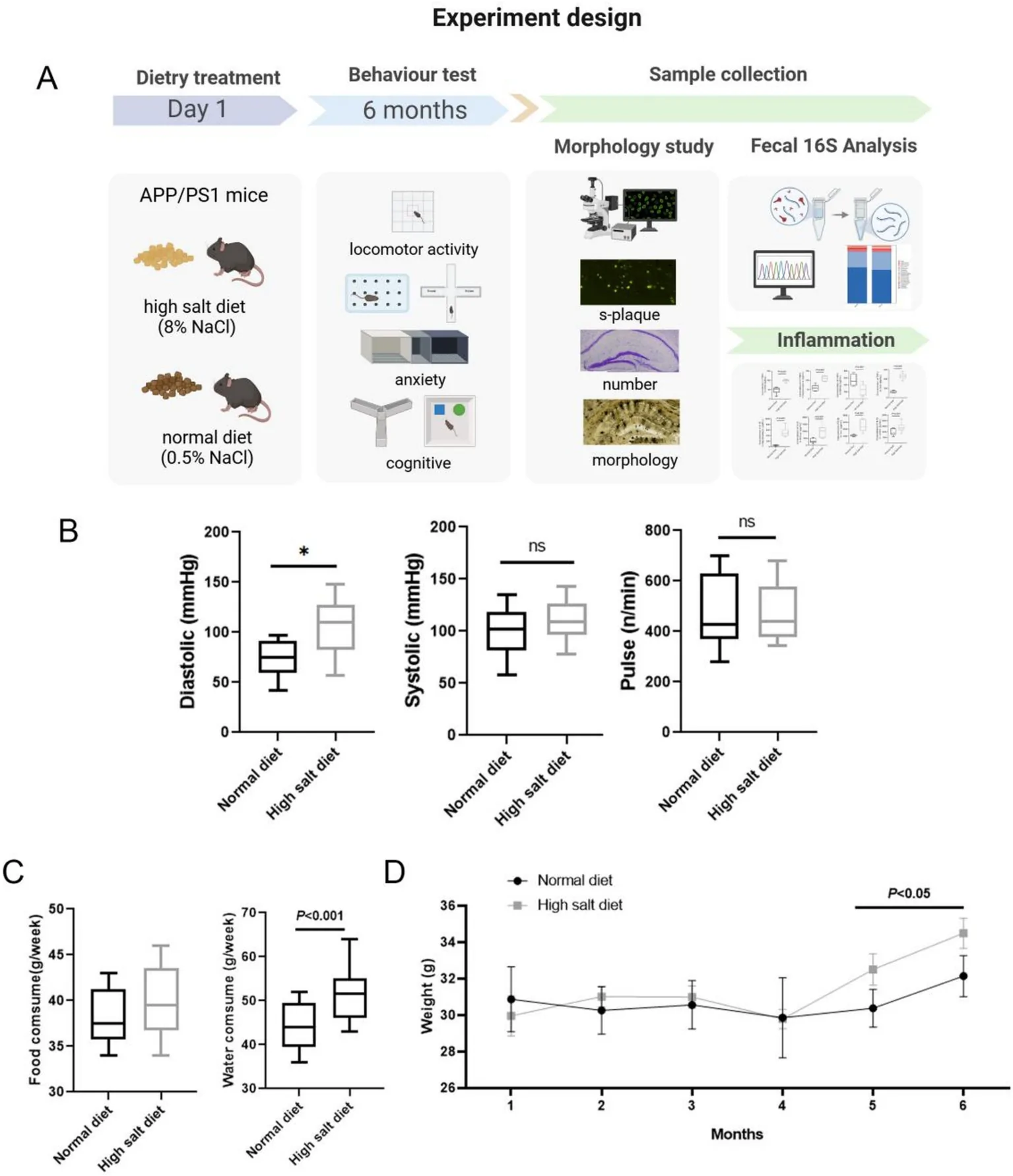
The effect of a high-salt diet on physiological indicators in mice **(A)** Schematic of experimental grouping and treatment. Mice were randomly divided into a normal diet group (ND, *n* = 10) and a high-salt diet group (HS, *n* = 10). Various tests were conducted after continuous feeding for 6 months. Using the *t*-test statistical method. **(B)** Blood pressure indicators. Compared with the normal diet group, the diastolic blood pressure of mice in the high-salt diet group was significantly elevated, while there was no significant difference in systolic blood pressure and pulse pressure. **(C)** Water intake and food intake. The water intake of mice in the high-salt diet group was significantly increased (*p* < 0.001), and there was no significant difference in food intake between the two groups. **(D)** Weight changes. Mice in the high-salt diet group had significantly lower body weights than those in the normal diet group at the 5th and 6th months of intervention (*p* < 0.05).

APP/PS1 mice received 6-month 8% NaCl high-salt or 0.4% normal-diet intervention (Figure 1A). Compared with controls, the high-salt group exhibited elevated diastolic blood pressure (*p* = 0.0175), unchanged systolic blood pressure and pulse pressure, comparable food intake, increased water intake (*p* < 0.001), and decreased body weight at months 5 and 6 (*p* < 0.05) (Figures 1B–D).

**Figure 2:**
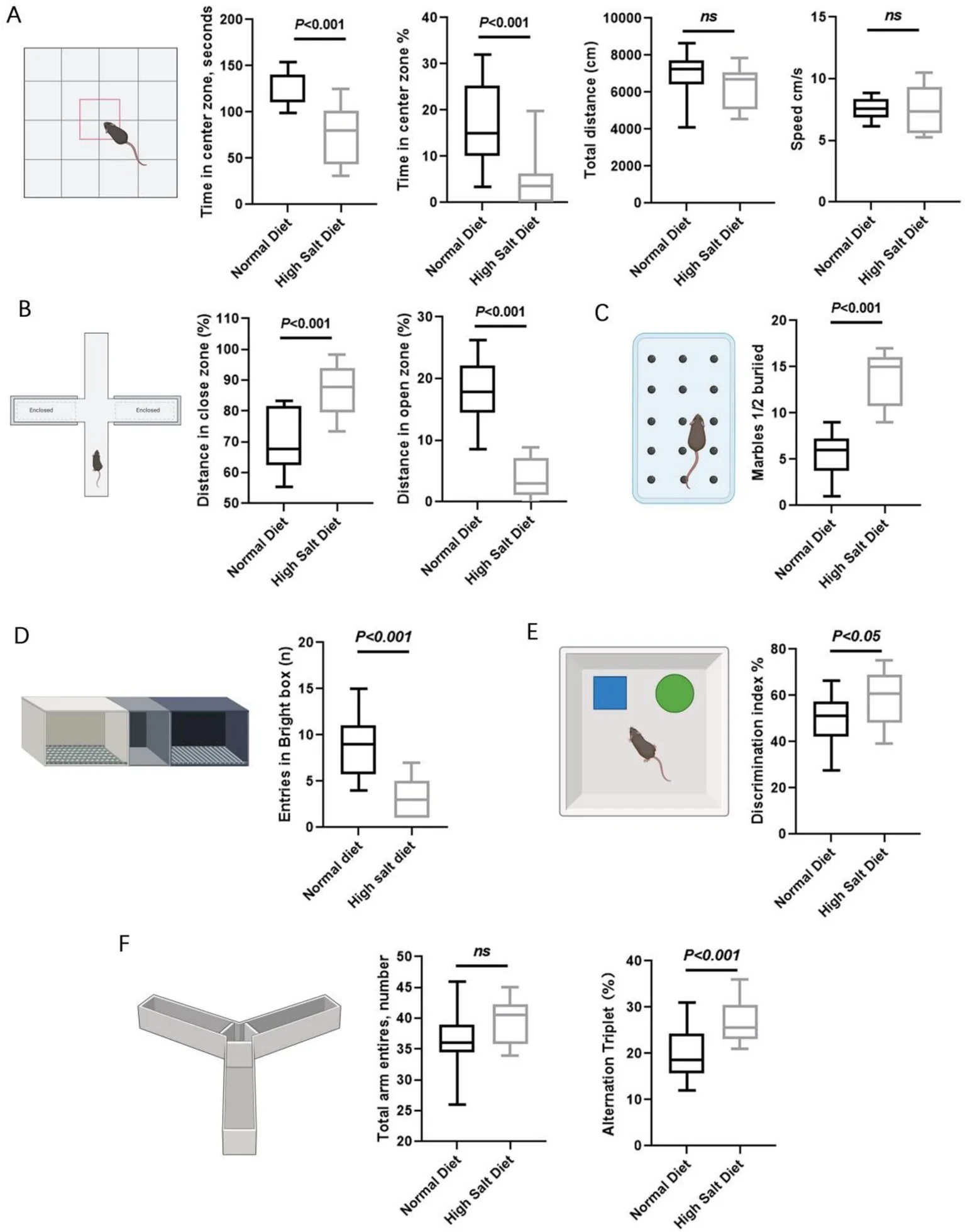
The effect of a high-salt diet on behavioral manifestations in mice. Mice were randomly divided into a normal diet group (ND, *n* = 10) and a high-salt diet group (HS, *n* = 10). After being fed for 6 months, various behavioral tests were conducted. The *t*-test was used as the statistical method. **(A)** Open-field test. Compared with the normal diet group, mice in the high-salt diet group spent significantly less time in the center zone (*p* < 0.001), while there were no significant differences in total movement distance and movement speed between the two groups (*p* > 0.05). **(B)** Elevated cross-maze. Mice in the high-salt diet group showed a significant increase in movement distance in the closed arms (*p* < 0.001) and a significant decrease in movement distance in the open arms (*p* < 0.001). **(C)** Bead-burying experiment. The number of beads buried by mice in the high-salt diet group (1/2 buried) was significantly higher than that in the normal diet group (*p* < 0.001). **(D)** In the black-and-white box experiment, mice in the high-salt diet group significantly reduced the number of times they entered the light box (*p* < 0.001). **(E)** Novel object recognition experiment. The discrimination index of mice in the high-salt diet group was significantly increased (*p* < 0.05). **(F)** Y-maze test. The number of three-arm alternations in the high-salt diet group mice was significantly increased (*p* < 0.001), while there was no significant difference in the total number of arm entries between the two groups.

Behavioural tests were performed 6 months after high-salt diet intervention to assess anxiety-related and cognitive phenotypes. In the 6-min open-field test, high-salt mice displayed reduced central-zone residence time (*p* < 0.001), with unchanged total distance and speed (Figure 2A). In the elevated plus-maze, high-salt mice traveled farther in closed arms (*p* < 0.001; Figure 2B). The marble-burying test revealed more buried marbles in the high-salt group (*p* < 0.001; Figure 2C). High-salt mice had fewer light-zone entries in the light–dark box test (*p* < 0.001; Figure 2D). Although the high-salt group showed elevated discrimination index in novel-object recognition (*p* < 0.05; Figure 2E) and higher Y-maze alternation with comparable total-arm entries (*p* < 0.001; Figure 2F), these changes cannot be interpreted as cognitive improvement. Instead, we propose that higher discrimination index and alternation rate reflect anxiety-mediated novelty-exploration bias across behavioral tasks.

### 3.2 High-salt diet induce neurodegeneration in the CA1 and CA2 of hippocampus in AD mice

**Figure 3:**
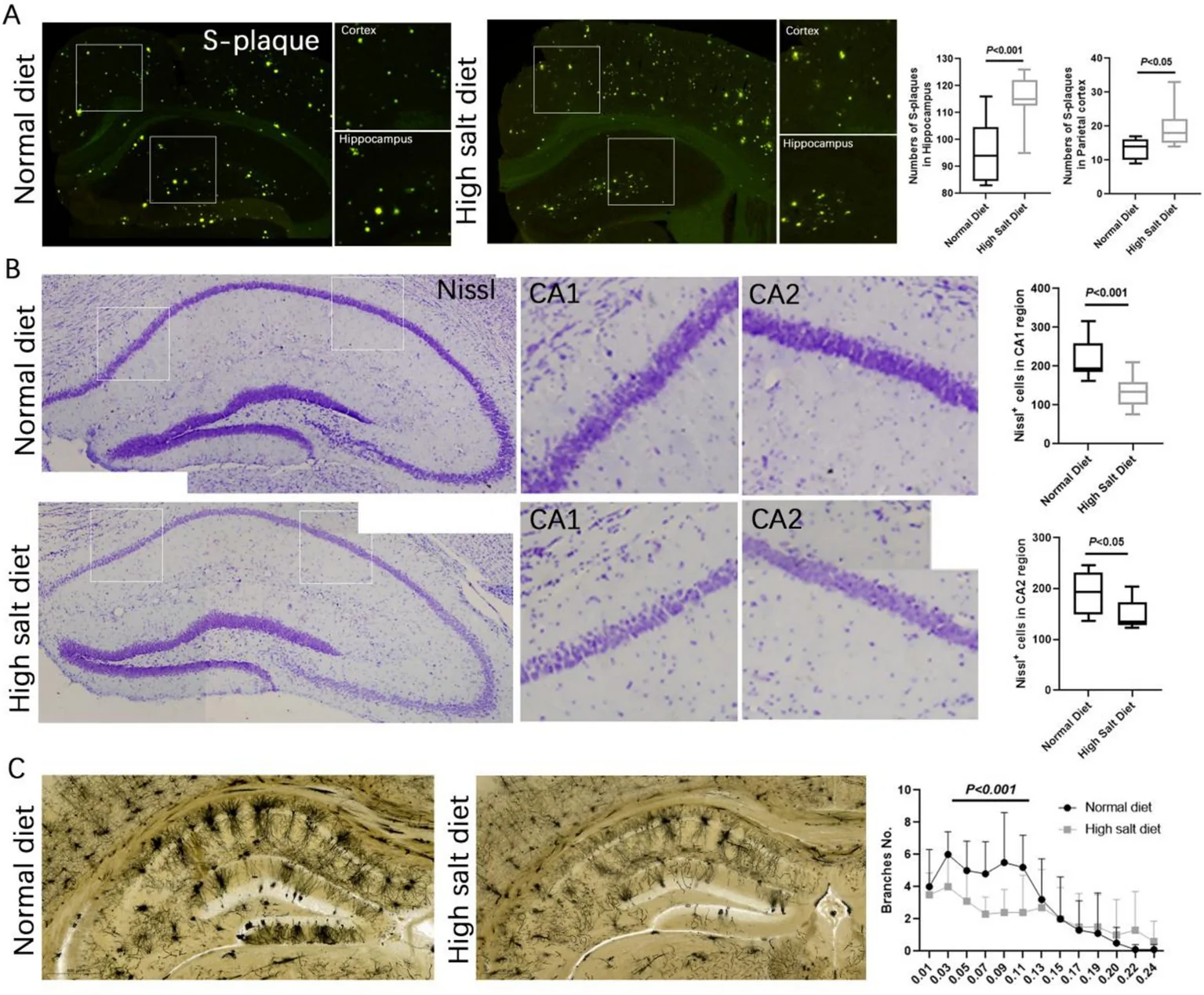
The effect of a high-salt diet on brain tissue pathology and neuronal morphology in APP/PS1 mice. Mice were randomly divided into a normal diet group and a high-salt diet group (*n* = 4). After continuous feeding for 6 months, tissue morphological examination was performed. The *t*-test statistical method was used. **(A)** Thiamine staining and S-plaque quantitative analysis. Compared with the normal diet group, the high-salt diet group showed a significant increase in the number of S-plaques in the hippocampus (*p* < 0.001) and also a significant increase in the number of S-plaques in the parietal cortex (*p* < 0.001). **(B)** Nissl staining and analysis of Nissl body density in the pyramidal cell layer. The high-salt diet group showed a significant decrease in Nissl body density in the pyramidal cell layer of the hippocampal CA1 region (*p* < 0.001), and a significant decrease in Nissl body density in the CA2 region as well (*p* < 0.05). **(C)** Golgi staining and dendritic spine morphological analysis. The high-salt diet group showed a significant decrease in dendritic spine density, indicating a significant reduction in the number of basal dendritic branches (*p* < 0.05).

High-salt diet group significantly affected the neural structure of the hippocampus and cortex in APP/PS1 mice. Number of S-plaques in the high-salt diet group significantly increased in the in the hippocampus and the parietal cortex (*p* < 0.001) (Figure 3A). The high-salt diet group showed a significant decrease in Nissl positive cells the hippocampal CA1 and CA2 region (*p* < 0.05) (Figure 3B). Sholl analysis showed that dendritic spine density was also decreased in the hippocampus, indicating a significant reduction in the number of basal dendritic branches (*p* < 0.05) (Figure 3C).

### 3.3 High salt affects the diversity and composition of gut microbiota in AD mice

**Figure 4:**
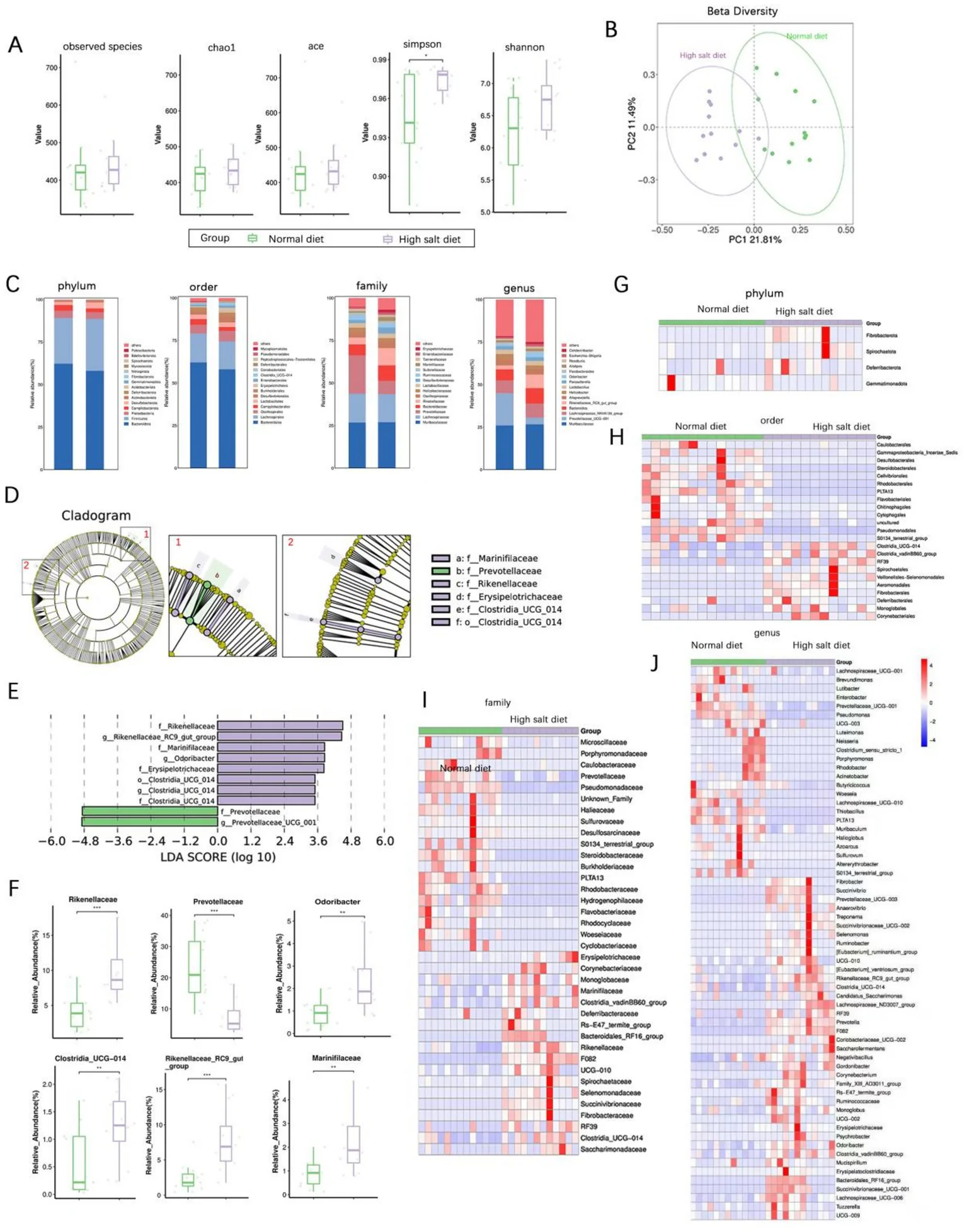
The effect of a high-salt diet on the gut microbiota of APP/PS1 mice. The gut microbiota of mice in the normal diet group (*n* = 13) and high-salt diet group (*n* = 12) were analyzed using 16S rRNA gene sequencing. **(A)** Alpha diversity analysis showed a significant increase in the Simpson index in the HSD group (*p* = 0.012). **(B)** Beta diversity analysis, including PCoA based on Bray-Curtis distance and Adonis test, revealed a significant separation of microbial community structures between the two groups (*p* = 0.001). **(C)** Species composition analysis showed that at the phylum level, the dominant bacteria were Bacteroidota, Firmicutes, and Proteobacteria. At the family level, the relative abundances were primarily dominated by Muribaculaceae, Lachnospiraceae, and Prevotellaceae. **(D–E)** LEfSe analysis and differential microbial analysis showed that the HSD group was enriched with Clostridia_UCG_014, Rikenellaceae, Erysipelotrichaceae, Marinifilaceae, Acholeplasmataceae, Rikenellaceae_RC9_gut_group, and *Odoribacter* at the order, family, and genus levels, while the ND group was enriched with Prevotellaceae. **(F)** Relative abundance comparisons of differential taxa at the family and genus levels. At the family level, Rikenellaceae and Marinifilaceae were enriched in the HSD group, while Prevotellaceae was enriched in the ND group. At the genus level, Rikenellaceae_RC9_gut_group, Odoribacter, and Clostridia were enriched in the HSD group. **(G)** Relative abundance comparisons of differential taxa at the phylum level. The abundances of Deferribacterota and Spirochaetota were significantly increased in the HSD group. **(H)** Relative abundance comparisons of differential taxa at the order level. Clostridia_UCG_014 was significantly enriched in the HSD group. **(I)** Relative abundance comparisons of differential taxa at the family level. The HSD group exhibited higher abundances of Rikenellaceae, Erysipelotrichaceae, Marinifilaceae, and Acholeplasmataceae, whereas Prevotellaceae was more abundant in the ND group. **(J)** Relative abundance comparisons of differential taxa at the genus level. Rikenellaceae_RC9_gut_group, Odoribacter, and Clostridia were enriched in the HSD group.

The α-diversity and β-diversity of the gut microbiota between two groups were analyzed using 16S rRNA gene sequencing (Figures 4A,B). The gut microbiota richness was increased in the high-salt diet group, with a significant difference in the Simpson index (Figure 4A). This may be due to changes in the intestinal environment, leading to the depletion of some bacteria and the expansion of salt-tolerant bacteria (Hamad et al., 2022). Meanwhile, beta diversity analysis (Adonis, PERMANOVA *p* = 0.001) showed significant changes in species composition in the high-salt diet group (Figure 4B). Which indicate that a high-salt diet alters the diversity and species composition of the gut microbiota. We further classified the differences in gut microbiota between the two groups. At the phylum, order, family and genus levels, the dominant phyla were *Bacteroidota*, *Firmicutes*, and *Proteobacteria*, with an increased ratio of Firmicutes to *Bacteroidota*. In the high-salt diet group, the abundances of *Deferribacterota* and *Spirochaetota* were significantly increased (Figures 4C,G). At the family level, the relative abundances were successively *Muribaculaceae*, *Lachnospiraceae*, *Prevotellaceae*, etc. (Figure 4C).

LEfSe analysis identified taxa that were differentially enriched between the two groups (Figures 4D,E), including taxa at the order, family, and genus levels. At the family level, the high-salt diet group was enriched in *Rikenellaceae*, *Erysipelotrichaceae*, *Marinifilaceae*, and *Acholeplasmataceae*, whereas *Prevotellaceae* was enriched in the normal diet group (Figures 4E,F,I). At the genus level, the high-salt diet group showed enrichment of *Rikenellaceae*, *Odoribacter*, and *Clostridia* (Figures 4E,F,H,J). Among these taxa, *Clostridia* and *Erysipelotrichaceae* belong to the phylum *Firmicutes*, whereas *Odoribacter* and *Rikenellaceae* belong to the phylum *Bacteroidetes*. *Acholeplasmataceae* belongs to the phylum *Tenericutes*.

### 3.4 High-salt diet affects the functional prediction of gut microbiota in AD mice

**Figure 5:**
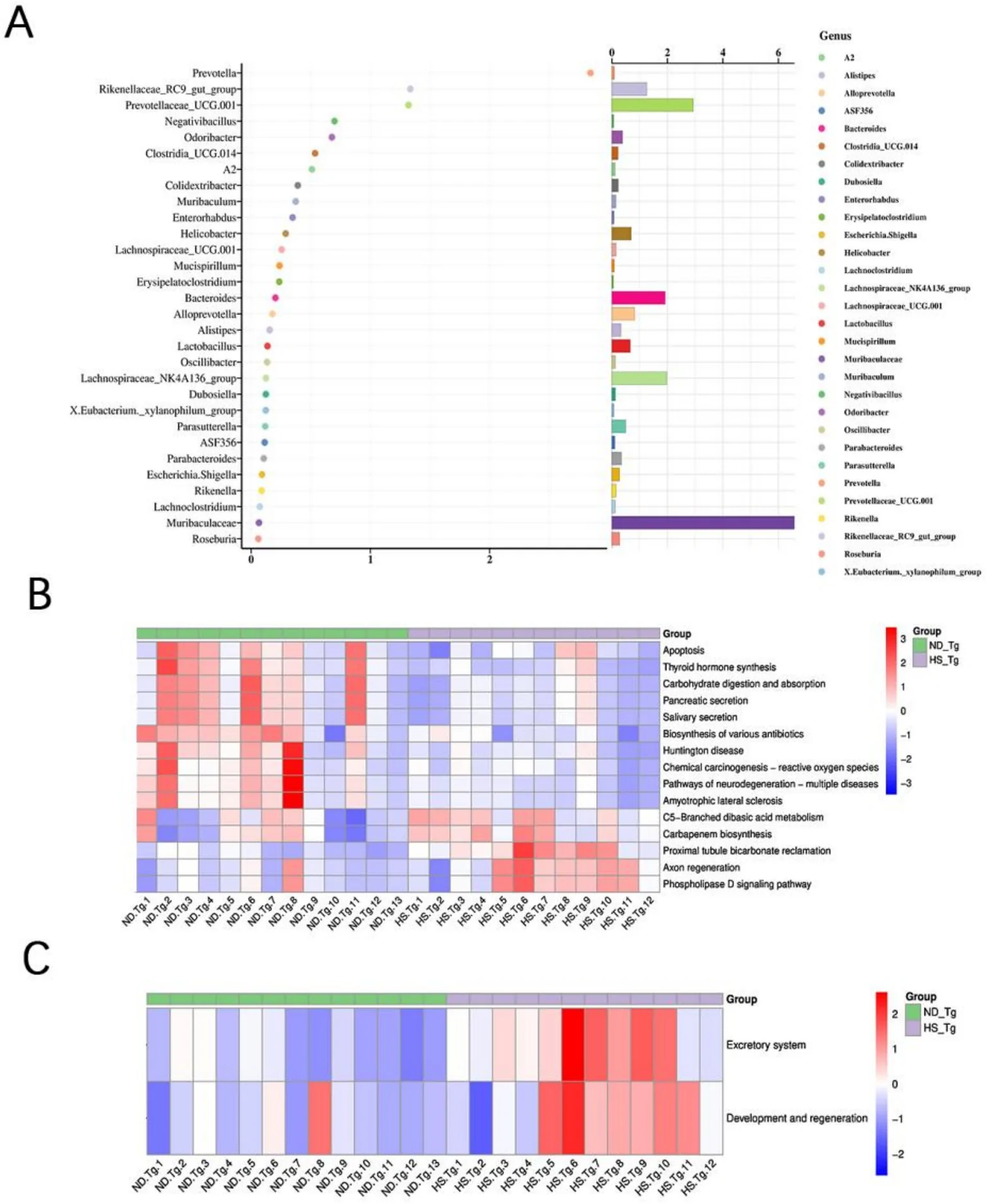
Model prediction and functional analysis based on gut microbiota. **(A)** Random forest analysis showed that Rikenellaceae_RC9_gut_group, *Odoribacter*, Clostridia_UCG_014, and Erysipelotrichaceae contributed more to the model prediction in the high-salt diet group, while Prevotellaceae_UCG_001 contributed more in the normal diet group. **(B)** KEGG Level 2 functional prediction showed significant differences between the two groups in the Excretory system (*p* = 0.00039) and Development and regeneration (*p* = 0.0195) pathways. **(C)** KEGG Level 3 functional prediction showed that the normal diet group had significantly increased expression in the Apoptosis (*p* = 0.0244) and Pathways of neurodegeneration – multiple diseases (*p* = 0.0083) pathways.

To investigate the effects of a high-salt diet on the gut microbiota, bacterial genera were analyzed using random forest analysis. The importance of high-abundance genera was ranked in descending order based on the overlap between significantly differential taxa identified by random forest and LEfSe analyses. *Rikenellaceae*, *Odoribacter*, *Clostridia*, and *Erysipelotrichaceae* were enriched in the high-salt diet group, whereas *Prevotellaceae* was most abundant in the normal diet group (Figure 5A).

Using 16S rRNA-based KEGG functional prediction with PICRUSt2, we found that a high-salt diet altered the predicted functions of the gut microbiota in AD mice at both KEGG Level 2 and Level 3. At KEGG Level 2, the high-salt diet group showed significantly higher enrichment in the excretory system (*p* = 0.00039) and development and regeneration (*p* = 0.0195) pathways (Figure 5B). At KEGG Level 3, the normal diet AD group showed significantly higher enrichment in apoptosis (*p* = 0.0244) and pathways of neurodegeneration-multiple diseases (*p* = 0.0083), whereas the high-salt diet group showed significantly higher enrichment in the axon regeneration pathway (*p* = 0.0195) (Figure 5C).

### 3.5 High-salt diet affects the cytokines and metabolisms expression in hippocampus of AD mice

**Figure 6:**
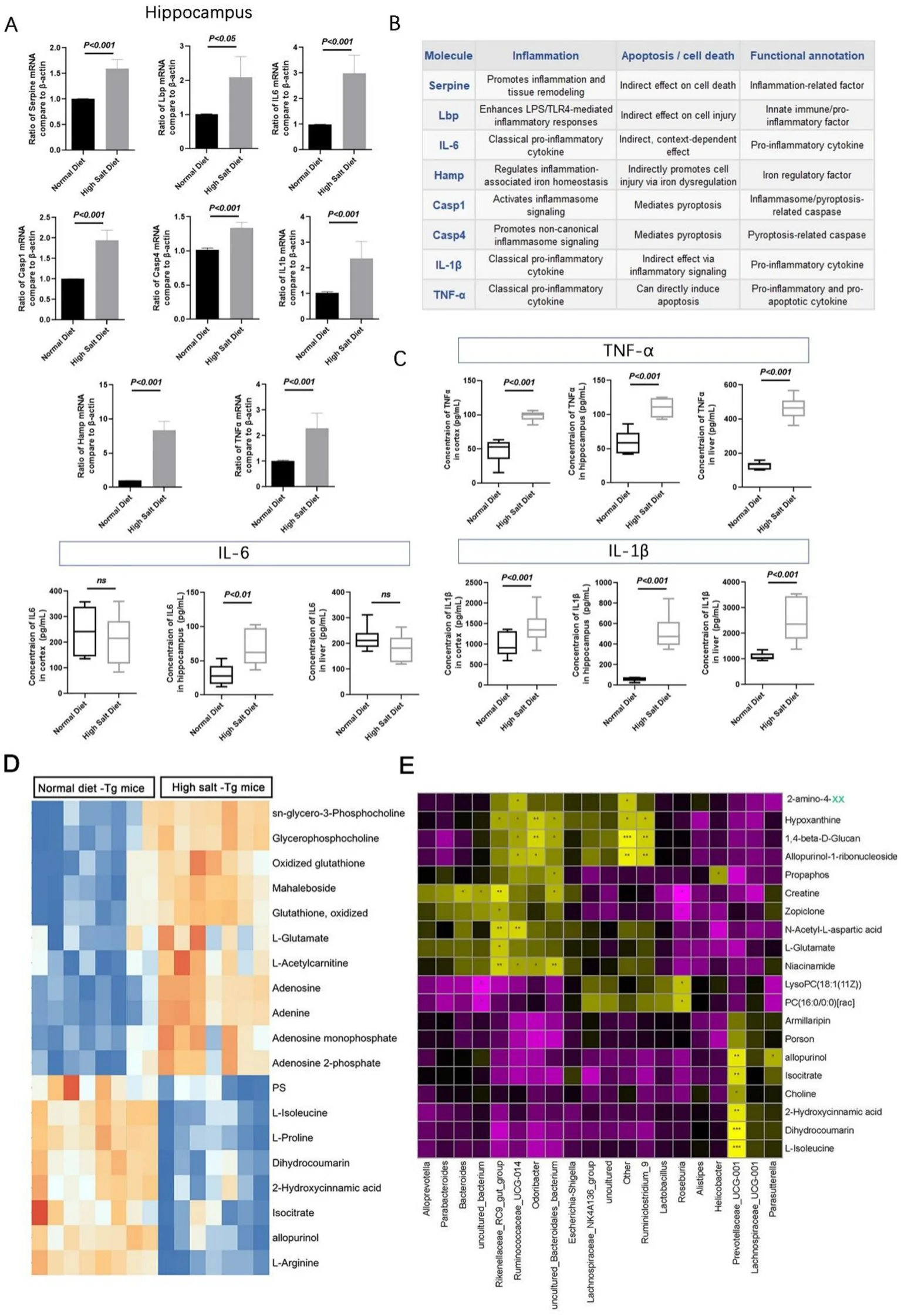
The effect of a high-salt diet on the expression of APP/PS1 mice neuroinflammation-related molecules. **(A)** qRT-PCR detection showed that the mRNA expression levels of Serpine, Lbp, IL-6, Casp1, Casp4, IL-1β, Hamp, and TNF-α in the brain tissues of mice in the high-salt diet group were significantly increased (*n* = 4, Lbp: *p* < 0.05; others: *p* < 0.001). **(B)** Molecular function annotations indicate that the above molecules primarily participate in inflammation response regulation, pyroptosis, and apoptosis processes (*n* = 4). **(C)** ELISA results showed that the concentration of TNF-α was significantly increased in both the cortex and hippocampus tissues of mice in the high-salt diet group (*n* = 4, *p* < 0.001); the concentration of IL-6 was significantly increased in the hippocampus tissue (*p* < 0.05); and the concentration of IL-1β was significantly increased in both the hippocampus and liver tissues (*p* < 0.001). **(D)** Metabolomic analysis revealed multiple differential metabolites between the two groups (*n* = 8 in normal diet group, *n* = 7 in high-salt diet group); **(E)** Correlation analysis metabolisms and differential taxa, yellow: Positively correlated, purple: Negatively correlated. “2-amino-4-XX” represents: 2-amino-4- ({1-[(carboxymethyl)-C-hydroxycarbonimidoyl]-2- ({4-[(8-{2-[(2-hydroxyacetyl)oxy]propan-2-yl}-2-oxo-2H,8H,9H-furo[2,3-h]chromen-9-yl)oxy]-3-methyl-4-oxobutan-2-yl}sulfanyl)ethyl}-C-hydroxycarbonimidoyl)butanoic acid.

To investigate the effect of a high-salt diet on neuroinflammation in APP/PS1 mice. We find that *Serpine*, *Lbp*, *IL-6*, *Casp1*, *Casp4*, *IL-1β*, *Hamp*, and *TNF-α mRNA* expression levels in high-salt diet group were significantly increased (*Lbp*: *p* < 0.05; others: *p* < 0.001) (Figure 6A). Molecular function annotation shows that these molecules are primarily involved in regulating inflammatory responses, pyroptosis, and apoptosis (Figure 6B). Among them, *TNF-α* was significantly increased in both the cortex and hippocampus tissues of mice in the high-salt diet group (*p* < 0.001); the concentration of *IL-6* was significantly increased in the hippocampus tissue (*p* < 0.05); and the concentration of *IL-1β* was significantly increased in both the hippocampus and liver tissues (*p* < 0.001) (Figure 6C).

Metabolomic analysis revealed multiple differential metabolites between the two groups, spanning amino acids, purines, organic acids, and phospholipids, indicating that a high-salt diet substantially reshaped the hippocampal metabolic profile. Notably, several of these differential metabolites were significantly correlated with the differential taxa. *Prevotellaceae* showed the most prominent associations: the branched-chain amino acid L-isoleucine, which is involved in protein synthesis and energy metabolism, was significantly positively correlated with this genus (*p* < 0.001), as were isocitrate, a key intermediate of the tricarboxylic acid cycle reflecting mitochondrial energy metabolism (*p* < 0.01), and 2-hydroxycinnamic acid and dihydrocoumarin, both of which possess antioxidant and anti-inflammatory properties (both *p* < 0.001), (Figure 6D).

In addition, L-glutamate, the major excitatory neurotransmitter in the central nervous system and closely related to learning and memory, was significantly positively correlated with *Rikenellaceae* and *Ruminococcaceae*. Furthermore, hypoxanthine, N-acetyl-L-aspartic acid, niacinamide, and creatine-metabolites involved in purine metabolism, neuronal metabolism, and energy metabolism-were significantly positively correlated with *Ruminococcaceae*, *Odoribacter*, and *Rikenellaceae* (Figure 6E).

## 4 Discussion

### 4.1 High-salt diet alters cognitive and anxiety like behavior in APP/PS1 mice

To support translational interpretation, mouse salt intake was converted to human equivalent dose (HED) using the FDA-recommended body surface area scaling method. According to feed salt concentration and average daily food consumption, the calculated human equivalent daily salt intake reached approximately 34.1 g for a 60 kg adult, which greatly exceeds the WHO recommended limit (<5 g/day) and the average intake (12–18 g/day) among northwest Chinese residents. This high-salt dietary intervention represents a classic and well-recognized mouse model to recapitulate human high-salt exposure. Consistent alterations in gut microbiota induced by high-salt diets have been documented in both human observational cohorts and mouse animal experiments. For instance, reductions in Lactobacillus abundance and impaired butyrate production are shared phenotypes across humans and mice under excessive salt exposure. Nevertheless, the salt threshold triggering microbial disturbance, the magnitude of community shifts, and strain-specific susceptibility to sodium stress are not identical.

In our study, APP/PS1 mice fed a high-salt diet exhibited specific alterations in behavioral and metabolic phenotypes. Mice in high-salt diet group showed a significantly increased water intake indicating that animals cope with high salt load through osmoregulatory mechanisms. The high-salt diet group showed more significant weight gain at months 5 and 6, which may be related to enhanced sympathetic nervous system activity (Brooks et al., 2005) and promotion of white adipose tissue hyperplasia and hypertrophy (Rahmouni et al., 2005). Meanwhile, its diastolic blood pressure remains persistently high, consistent with the hemodynamic changes associated with high sodium intake in population studies (Brooks et al., 2005). This indicates that a high-salt diet triggers significant metabolic and physiological changes by affecting osmoregulation, sympathetic nervous system activity, and fat metabolism. In terms of cognitive behavior, high-salt diet exacerbates anxiety-like behaviors in APP/PS1 mice, impairs cognitive function, and increases stereotypical behaviors.

However, in the Y-maze test, the high-salt diet group showed an increased number of alternating explorations, contrary to expectations. In the presence of a pronounced anxiety-like phenotype, cognitive assessments based on spontaneous exploration may not accurately reflect true memory ability, but may instead be confounded by the animal’s emotional state. This consideration has been clearly articulated in previous methodological studies (Bevins and Besheer, 2006; Blaser and Heyser, 2015). More directly, Ennaceur et al., demonstrated that in an anxiety state, cognitive processes are overwhelmed by numerous indiscriminate stimuli, and the difference between objects becomes less important. This may stem from chronic stress caused by long-term high-salt diet altering exploration strategies (Lu et al., 2021).

On the basis of the above literature, we reason that in the high-salt group of the present study—which displayed a significant and consistent anxiety-like phenotype, the exploration-based cognitive indices from the NOR and Y-maze tests were most likely confounded by anxiety, and therefore cannot be directly or reliably interpreted as a genuine improvement in cognitive function. This stress may trigger hyperactive or disorganized behavioral patterns, leading to an increased number of arm alternations; therefore, this increase may not be interpreted as an improvement in memory, but rather as a manifestation of behavioral disorganization (Cinque et al., 2018). Taken together, we interpret the increased discrimination index and alternation rate not as enhanced cognition, but as a coherent, cross-task manifestation of an anxiety-driven novelty-exploration bias.

### 4.2 High-salt diet leads to neuronal loss and inflammation in APP/PS1 mice

High-salt diet is associated with impaired cognitive function and mood changes, which may damage memory by interfering with hippocampal synaptic plasticity (Ge et al., 2017). Chronic high-salt diets can lead to permanent neuronal damage (Meissner et al., 2022). In our 6-month animal experiment, a high-salt diet significantly increased S-plaque deposition in the hippocampus and cortex, reduced Nissl body density in the hippocampal CA1 and CA2 regions, and decreased dendritic spine density and basal dendritic branching. Which exhibits the upregulated neuroinflammation and oxidative stress (Chen H. C. et al., 2024), neuronal loss (Liu et al., 2014) and synaptic plasticity impairment (Yang X. et al., 2024).

High-salt diet damages the intestinal barrier and increases its permeability, allowing bacterial products to enter the bloodstream and trigger systemic inflammation. This, in turn, promotes the entry of inflammatory factors into the brain, inducing neuroinflammation (Leblhuber et al., 2021; Peddinti et al., 2024). The results of this study indicate that a high-salt diet not only induces local central nervous system inflammatory responses and accelerates plaque formation but may also trigger a systemic inflammatory state through peripheral-central interactions. As key effector molecules of pyroptosis, the significant upregulation of Casp1 and Casp4 suggests that a high-salt diet may activate both classical and non-classical inflammasome pathways, thereby promoting the maturation and release of pro-inflammatory cytokines such as IL-1β (Liu et al., 2014). Combining S-plaque deposition and neuronal structural damage, this study suggests that high-salt diet may synergistically drive neuroinflammatory responses and cell death through pyroptosis mechanisms mediated by inflammasomes, providing important molecular mechanism clues for central nervous system pathological changes.

In line with the systemic nature of salt stress, our data showed markedly elevated renal pro-inflammatory cytokine levels in the high-salt group, confirming that a high-salt diet induces renal inflammation. This observation is consistent with the finding that, at physiologically relevant concentrations, sodium chloride promotes the induction of pathogenic Th17 cells through the p38/MAPK–NFAT5–SGK1 pathway and upregulates pro-inflammatory cytokines including GM-CSF, TNF-α, and IL-2, with high-salt–fed mice exhibiting more severe inflammation and enhanced central nervous system infiltration (Kleinewietfeld et al., 2013). Thus, high salt may drive concurrent renal and neural inflammation via a shared pro-inflammatory Th17-mediated mechanism. Importantly, renal injury and the gut microbiota are reciprocally linked: the uremic state has been shown to profoundly alter gut microbial composition, with significant shifts in numerous bacterial taxa (e.g., reduced Lactobacillaceae and Prevotellaceae) (Vaziri et al., 2013), indicating that impaired renal function can in turn reshape the gut microbiota through a bidirectional gut–kidney axis. Together with the high-salt–induced microbiota alterations observed here, these findings suggest that renal inflammation and gut dysbiosis may mutually reinforce one another under salt stress, and, alongside the accompanying neuroinflammation and cognitive changes, point to a coordinated gut–kidney–brain crosstalk through which high salt exerts its multi-organ effects.

### 4.3 High-salt diet alters the composition of the gut microbiota

Long-term high-salt diet induces gut microbiota dysbiosis and impairs intestinal barrier function, thereby exacerbating neuroinflammation in AD mice. Our findings showed that *Bacteroidota*, Firmicutes, and Proteobacteria were the dominant phyla, and that the increased *Firmicutes*/*Bacteroidota* ratio is generally considered indicative of substantial changes in gut microbiota structure and composition, that is, gut dysbiosis (Wu et al., 2023; Shon et al., 2024). Gut microbiota dysbiosis may regulate neuroinflammation by altering intestinal permeability, affecting microbial metabolites, and regulating the immune system (Das and Ganesh, 2023; Ngah et al., 2024; Ajith and Sreejith, 2025; Palanivelu et al., 2025). LEfSe analysis showed that under long-term high-salt diet conditions, the abundance of *Rikenellaceae* increased. This is an important member of the Bacteroidetes phylum, its change exhibits gut microbiota imbalance (Yang J. et al., 2024). We also observed an increased abundance of *Clostridia* in the gut microbiota under high-salt diet treatment, which may indicate a transition of the intestinal microecology from symbiosis to dysbiosis (Nikola and Iva, 2024). There is currently very limited studies on the relationship between the *Marinifilaceae* family and Alzheimer’s disease. Therefore, it may indirectly affect the host’s health status in the intestinal microecosystem, thereby influencing the occurrence and development of AD (Doifode et al., 2021). Furthermore, the abundance of *Prevotellaceae* was significantly higher in the normal diet group than in the high-salt group, suggesting that increased *Prevotellaceae* may help maintain intestinal barrier function and immune balance (Precup and Vodnar, 2019).

### 4.4 High-salt diet alters the renal metabolic system

In the present study, we detected significantly elevated levels of classical pro-inflammatory cytokines in the kidneys of the high-salt group, confirming that a high-salt diet induces renal inflammation. This finding is consistent with previous work (Kleinewietfeld et al., 2013).demonstrated that, at physiologically relevant concentrations *in vivo*, sodium chloride (NaCl) markedly promotes the induction of pathogenic Th17 cells via activation of the p38/MAPK–NFAT5–SGK1 pathway, upregulating pro-inflammatory cytokines such as GM-CSF, TNF-α, and IL-2; moreover, mice fed a high-salt diet developed more severe inflammation and enhanced central nervous system infiltration. This indicates that high salt can drive multi-organ inflammation—including in the kidney—through pathogenic Th17 responses and pro-inflammatory cytokine production, thereby linking the renal inflammation observed in our study with neuroinflammation and cognition-related changes. Furthermore, a close bidirectional relationship exists between renal injury and the gut microbiota. (Vaziri et al., 2013), analyzing patients with end-stage renal disease (ESRD) and a 5/6-nephrectomy uremic rat model, found that the uremic state profoundly alters the composition of the gut microbiota, with significant changes in the abundance of numerous bacterial operational taxonomic units (OTUs) (e.g., decreased *Lactobacillaceae* and *Prevotellaceae*).

### 4.5 Gut dysbiosis participates in high-salt diet-induced inflammation

High-salt diet exacerbating neuroinflammation and systemic inflammatory responses may through disruption of the intestinal barrier, intestinal permeability and dysbiosis (Zhang et al., 2023). And then the bacterial products and pro-inflammatory molecules enter into the bloodstream, triggering a systemic inflammatory response, such as chronic brain inflammation (Leblhuber et al., 2021; Peddinti et al., 2024). Our study shows that at the phylum level, the abundance of *Deferribacterota* is significantly increased in the high-salt diet group. Which is a class of anaerobic or microaerophilic Gram-negative bacteria, have low abundance in the healthy gut environment, is upregulated in high salt treated mice (Saqib et al., 2023). At the family level, there is a significant change in the abundance of *Acholeplasmataceae* in AD patients. *Acholeplasmataceae* can influence neuroinflammation by affecting intestinal barrier function or producing specific metabolites (Yang J. et al., 2024). In our study, at the Level 2 of KEGG functional prediction, significant differences were observed between the normal group and the high-salt group in the excretory system. High-salt diet exacerbates renal inflammatory responses and promotes the infiltration and activation of pro-inflammatory macrophages in renal tissues (Fagunwa et al., 2024). C*lostridia*, *Rikenellaceae*, and *Erysipelotrichaceae* are closely associated with the *Odoribacter* genus and the expression of pro-inflammatory cytokines TNF-α, IL-1β, and IL-6 (Dong et al., 2022; Fang et al., 2023; Kalashnikova et al., 2024). In summary, high-salt diets affect inflammatory status by disrupting the intestinal barrier and inducing dysbiosis.

High-salt diet-induced neuroinflammation may be a major driver of aggravated cognitive and emotional impairment. The inflammatory factors produced can cross the blood–brain barrier, triggering neuroinflammation, which in turn exacerbates neuronal damage and leads to cognitive impairment (Xu et al., 2025). We found that an increase in the abundance of Spirochaetota is potentially causally associated with neurocognitive dysfunction. The enrichment of Spirochaetota promotes the activation of pro-inflammatory pathways and weakens the production of anti-inflammatory and neuroprotective metabolites, ultimately leading to a comprehensive decline in cognitive function (Chen H. C. et al., 2024). Odoribacter belongs to the phylum Bacteroidetes. A high-salt diet can influence butyrate metabolism by altering its abundance, thereby regulating host intestinal health and cognitive function (Lei et al., 2023). The abundance of the family Acholeplasmataceae influences the progression of Alzheimer’s disease (Choi and Mook-Jung, 2023).

### 4.6 High-salt diet is associated with gut dysbiosis and neurodegeneration

At level 3 of functional prediction, apoptosis and pathways of neurodegeneration involving multiple diseases were significantly enriched in AD mice fed a normal diet, whereas axon regeneration was significantly enriched in mice fed a high-salt diet. These results suggest that high-salt intervention in AD mice differ greatly from those of wild-type mice. Regarding apoptosis, the decline from initially elevated to lower apoptotic levels under long-term high-salt stimulation may reflect a dynamic process of “elimination–adaptation–homeostasis.” The early high apoptotic rate reflects the direct consequence of AD pathology-induced damage, while the later low apoptotic rate serves as a marker of successful adaptation and establishment of a new tolerant homeostasis by the cell population (Relyea et al., 2023). A relative reduction in signaling related to pathways of neurodegeneration-multiple diseases was observed; however, this does not necessarily indicate that these pathways are truly inactive. Rather, it may reflect the overwhelming dominance of high-salt-induced, Th17-mediated neuroinflammation, which masks the phenotypic manifestation of classical neurodegenerative pathology (Zhao, 2024). Increased axon regeneration may be attributed to high salt, which activates neuronal Na^+^ channels, induces the formation of c-Fos/c-Jun AP-1 complexes, and upregulates the expression of the cytoskeletal protein βIII-tubulin, thereby promoting axonal sprouting and regeneration (Martín-Hersog et al., 2024).

Moreover, we find that several metabolites, which exhibited both intergroup differences and associations with the gut microbiota, were primarily enriched in amino acid metabolism, energy metabolism, and antioxidant pathways, suggesting that a high-salt diet may influence hippocampal metabolic processes by modulating specific taxa, thereby providing molecular-level evidence for the link between the gut microbiota, metabolism, and hippocampal function.

## 5 Conclusion

Chronic high-salt intake was associated with increased anxiety-like behaviors, gut microbiota dysbiosis, an elevated *Firmicutes*/*Bacteroidetes* ratio, and shifts in specific microbial taxa. Among these, *Rikenellaceae*, *Erysipelotrichaceae*, and *Odoribacter* may be linked to increased hippocampal inflammation and neuronal damage. These results support a potential role for the gut microbiota–hippocampal inflammation axis in mediating the effects of a high-salt diet on AD-related pathology, and provide a basis for further exploration of dietary interventions and microbiota-targeted strategies in AD. However, it has several limitations. First, although we identified significant correlations between specific gut microbial taxa, hippocampal metabolites, and inflammatory outcomes, these relationships remain correlational rather than causal, and the individual steps of the microbiota–metabolism–brain axis were not directly validated. Second, gut barrier permeability and intestinal histopathology were not measured, and the PICRUSt2-based functional data are predictive in nature and not equivalent to direct measurements of microbial metabolites or pathway activity; future work incorporating gut barrier assessment and direct functional/causal validation is warranted. It can be concluded that high-salt diet may exacerbate Alzheimer’s disease pathology by altering the gut microbiota and enhancing hippocampal pro-inflammatory cytokine expression.

## Data Availability

The data analyzed in this study are available in the NCBI SRA under accession number PRJNA1520793: https://www.ncbi.nlm.nih.gov/sra/PRJNA1520793.
